# Chemotherapy drugs cyclophosphamide, cisplatin and doxorubicin induce germ cell loss in an *in vitro* model of the prepubertal testis

**DOI:** 10.1038/s41598-018-19761-9

**Published:** 2018-01-29

**Authors:** Ellie Smart, Federica Lopes, Siobhan Rice, Boglarka Nagy, Richard A. Anderson, Rod T. Mitchell, Norah Spears

**Affiliations:** 1 Biomedical Sciences, University of Edinburgh, Edinburgh, EH8 9XD United Kingdom; 2 MRC Centre for Reproductive Health, University of Edinburgh, Edinburgh, EH16 4TJ United Kingdom; 3 Present Address: Center for Dermatology Research, University of Manchester, Manchester, United Kingdom; 4 Present Address: Weatherall Institute of Molecular Medicine, MRC Molecular Haematology Unit, University of Oxford, Oxford, OX3 9DS, United Kingdom

## Abstract

Long term survival rates for childhood cancers is steadily increasing, however cancer survivors can experience fertility problems as a consequence of chemotherapy treatment. This is particularly problematic for young boys, for whom no fertility preservation treatment is yet established. Here, we have determined the effects on prepubertal mouse testis of three commonly used chemotherapy drugs; cyclophosphamide (using its active metabolite phosphoramide mustard), cisplatin and doxorubicin, exposing testicular fragments to a clinically relevant range of concentrations *in vitro*. All three drugs induced a specific and highly significant loss of germ cells, including spermatogonial stem cells. In contrast, there was no significant effect on somatic cells, for either Sertoli or interstitial cells. Time course analysis of cleaved Caspase-3 expression showed a significant increase in apoptosis eight hours prior to a detectable decrease in germ cell numbers following exposure to phosphoramide mustard or cisplatin, although this pattern was not seen following doxorubicin-exposure. Moreover, analysis of DNA damage at 16 h showed increased γH2AX expression in response to all three drugs. Overall, results show that cisplatin, doxorubicin and cyclophosphamide all specifically induce loss of germ cells, including of spermatogonial stem cells, in the prepubertal mouse testis at concentrations relevant to human therapeutic exposures.

## Introduction

Survival rates from most childhood cancers have increased dramatically over recent decades, with chemotherapy a mainstay of treatment for the majority of these patients. Ideally, chemotherapeutic agents would specifically target cancerous cells without affecting healthy tissue, however in practice there are many off-target effects of cytotoxic treatments, including on the gonads. There is now a growing population of cancer survivors suffering from chemotherapy-induced infertility with some also affected by hypogonadism, effects that can significantly impact on quality of life. The situation is of particular concern for male childhood cancer patients due to the lack of fertility preservation methods available for prepubertal males^1^.

Prepuberty in humans can be defined as the period from late infancy until the onset of puberty: during this period there is relatively little testicular activity, with low rates of proliferation within the spermatogonial germ cell and Sertoli cell populations compared to the frequent proliferation that occurs in these cells during infancy and adulthood^2,3^. Although occasional germ cells enter meiosis during prepuberty, these cells subsequently undergo apoptosis^4^. Of particular importance during the prepubertal period is the quiescence of the HPG axis with very low levels of gonadotrophins and testosterone^2,3,5^. The comparative quiescence of the prepubertal testis was once thought to confer protection from chemotherapy-induced damage^6^. However, males treated before puberty may present in adulthood with oligo- or azoospermia, with the extent of damage to the seminiferous epithelium related to both drug regimen and cumulative dose received^7^. Such damage might be related to the activity that takes place in the prepubertal testis, such as Sertoli cell maturation or germ cell proliferation^4,8^. Despite the clear evidence that chemotherapy drug exposure to prepubertal boys can affect subsequent fertility^9^, there is little information as to how these drugs damage the prepubertal testis. Given that the prepubertal testis tissue, germ cell stages and hormonal environment is markedly different from that of the pubertal or adult testis, it cannot be assumed that drug effects will be similar at both stages. Thus, a fuller understanding of how germ cells in the prepubertal testis are lost or damaged by specific chemotherapy drugs is vital for the provision of accurate advice and for the development of preventative treatments to protect fertility in these patients. It is also important to determine effects of these drugs specifically on the stem germ cell population, since survival of a sufficient number of spermatogonial stem cells (SSCs) allows for the possibility of subsequent germ cell repopulation of the testis, with the potential to restore fertility.

Here, we examine the individual effects of three different chemotherapeutic agents: cyclophosphamide (CYP); cisplatin (CIS); and doxorubicin (DOX). These three drugs were chosen as they are all commonly used in the treatment of a range of prepubertal cancers, thus with more clinical information available, including that of patient serum concentrations. CYP is an alkylating agent that is metabolised in the liver to a number of compounds, the most active of which is phosphoramide mustard (PM). PM binds an alkyl molecule to DNA, forming an adduct that prevents DNA replication^10^. It is widely believed that alkylating agents are the most gonadotoxic chemotherapy drugs, and that the degree of gonadal damage can be related to the dose of alkylating agent received^11^. CIS is one of several platinum-based chemotherapy drugs, all considered to act in an alkylating-like manner, but in which it is the platinum core that binds to DNA to form the adducts^12^. The action of the anthracycline DOX is somewhat different, as it intercalates with DNA, inhibiting Topoisomerase II and hence preventing replication^13^. DOX is often considered to be the least gonadotoxic of the agents examined here, only causing prolonged azoospermia when combined with other drugs^14^.

We aimed to determine the effects of exposure to each of PM, CIS or DOX on prepubertal testis within a clinically-relevant range of concentrations. We utilised an *in vitro* culture system of prepubertal mouse testis tissue which we have previously demonstrated to support short-term development of the prepubertal mouse testis^15^. This stage of development in rodents has important similarities to the human during prepuberty in terms of relative quiescence of the hypothalamic-pituitary-gonadal (HPG) axis and the presence of a germ cell population consisting almost exclusively of spermatogonia^4,5^. However, when determining potential relevance to humans there are also key species differences that should be considered such as the precise spermatogonial sub-populations and rates of Sertoli cell proliferation^3,4,16^. Using this system we were able to examine the direct effects of each drug, and to provide a comparison of relative gonadotoxicity between drugs. Exposure to each of the three chemotherapeutic agents resulted in a significant reduction in germ cell number, which include the promyelocytic leukemia zinc-finger-positive (PLZF^+^) SSC sub-population.

## Results

### Cyclophosphamide, cisplatin and doxorubicin each result in a specific loss of germ cells

**Figure 1:**
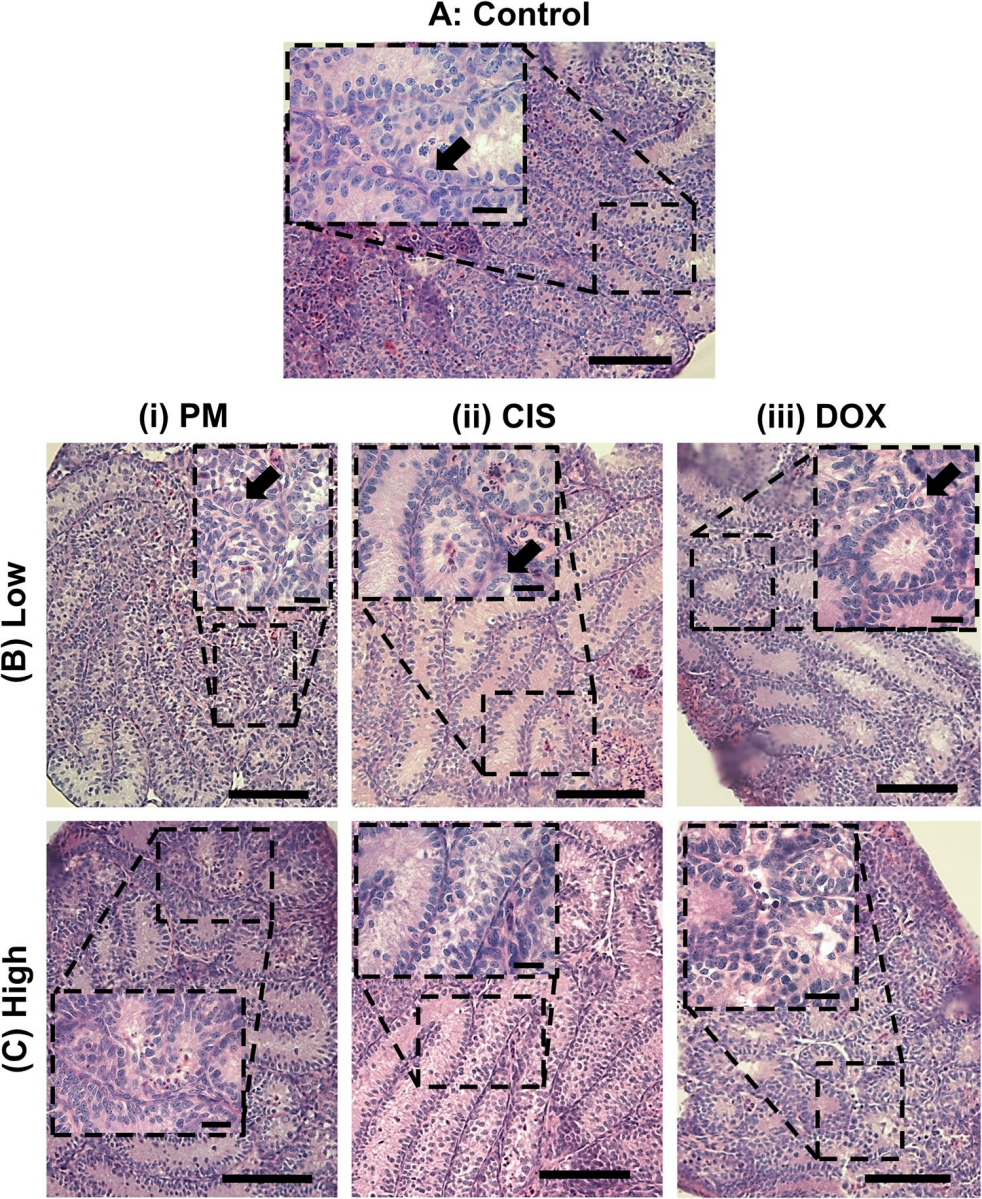
Effect of exposure to phosphoramide mustard, cisplatin or doxorubicin on tissue morphology. Representative photomicrographs of cultured testis fragments stained with haematoxylin and eosin. (**A**) Control tissue, or after exposure to (**B**) Low or (**C**) High concentrations of (i) PM, (ii) CIS or (iii) DOX. Arrows indicate germ cells. Scale bars represent 100 µm; scale bars in insets represent 20 µm.

**Figure 2:**
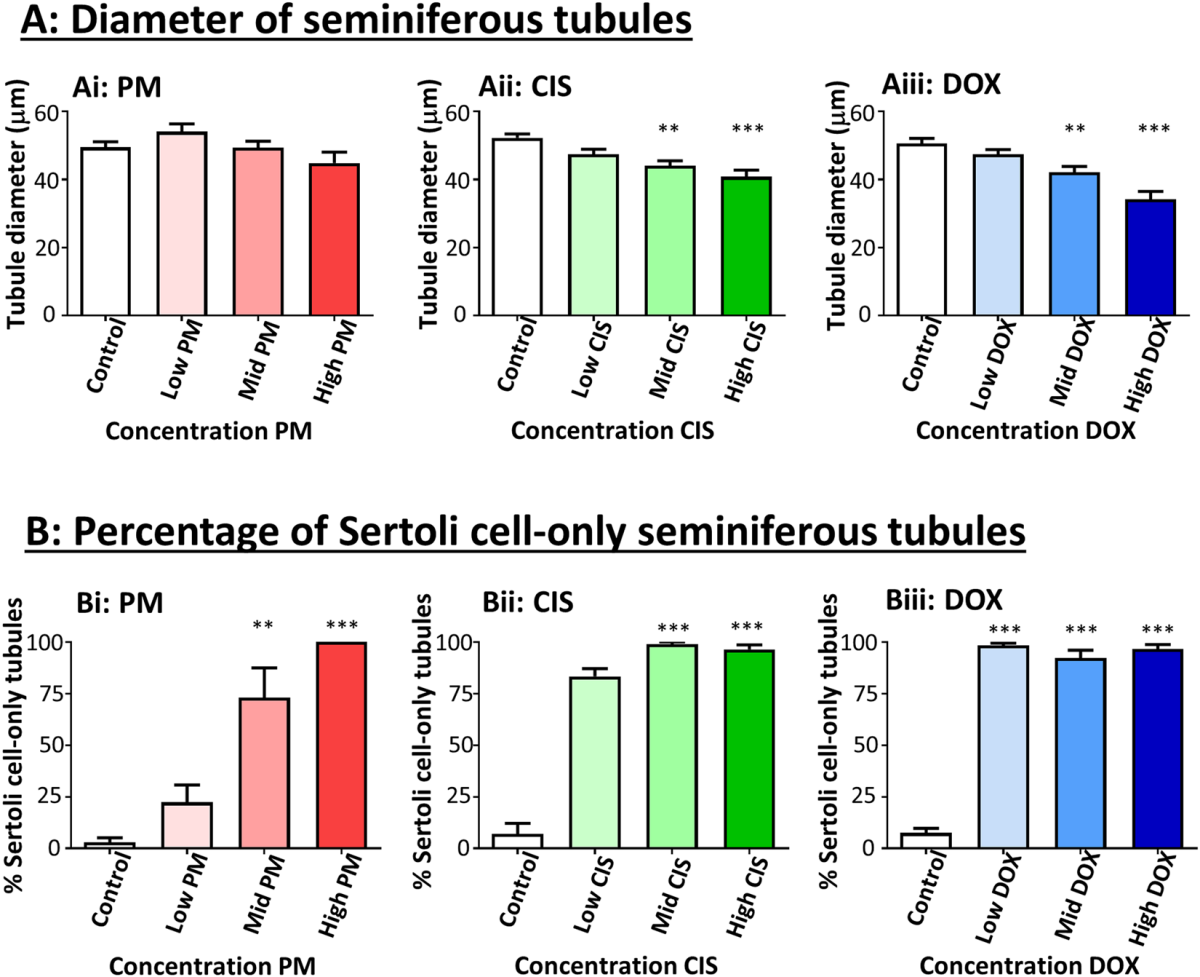
Exposure to phosphoramide mustard, cisplatin or doxorubicin results in smaller, and Sertoli cell-only seminiferous tubules. (**A**) Seminiferous tubule diameter; n = 8 for all conditions except high PM where n = 7. (**B**) Percentage of seminiferous tubules that contain only Sertoli cells: (Bi) PM; n = 6–17, (Bii) CIS; n = 5–8, (Biii) DOX; n = 11–17. Data are mean + SEM; p < 0.01 (**), p < 0.001 (***) for treatment versus Control.

Both Control testis and tissue exposed to PM, CIS or DOX had morphologically normal tubules, with basement membrane separating tubules from interstitium (Fig. 1). Control cultured testis was healthy, with germ cells situated along the basement membrane of the seminiferous tubules. After exposure to Low concentrations of PM, CIS or DOX, germ cells could still be observed either at the basement membrane or in the centre of the tubules (Fig. 1B); in contrast, it was difficult to identify germ cells after exposure to High concentrations of any of the three drugs, after which pyknotic cells were clearly visible and the majority of tubules appeared to contain only Sertoli cells (Fig. 1C). Seminiferous tubule diameter decreased in response to Mid (p < 0.01) or High (p < 0.001) levels of CIS or DOX, although no decrease was found after exposure to PM (Fig. 2A). Seminiferous tubules containing only Sertoli cells were found in less than 10% of Control cultured tubules, but these Sertoli cell-only tubules increased markedly in response to all three drugs, until over 95% of tubules lacked germ cells after exposure to the High concentrations of each drug (p < 0.001 for all drugs: Fig. 2B).

**Figure 3:**
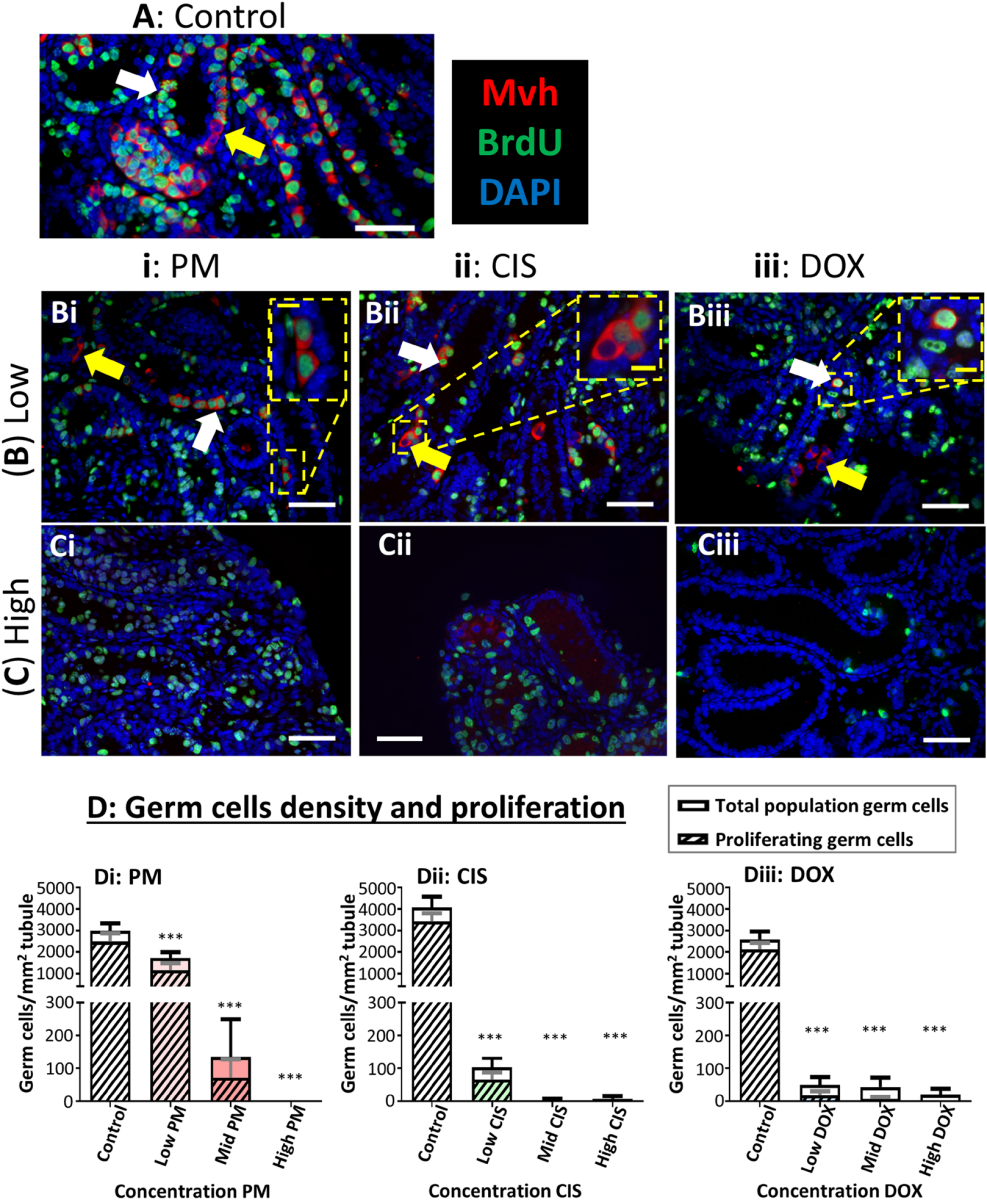
Exposure to phosphoramide mustard, cisplatin or doxorubicin results in germ cell loss. (**A–C**) Representative photomicrographs of cultured testis fragments showing immunohistochemical localisation of Mvh (red) and BrdU (green), counterstained with DAPI (blue) for (**A**) Control tissue, or after exposure to (**B**) Low or (**C**) High concentrations of: (i) PM, (ii) CIS or (iii) DOX. White arrows indicate Mvh^+^/ BrdU^+^ proliferating germ cells; yellow arrows indicate Mvh^+^/BrdU^−^ non-proliferating germ cells. (**D**) Number of Mvh^+^ germ cells (open bars) and Mvh^+^/ BrdU^+^ proliferating germ cells (hatched bars) per mm^2^ of seminiferous tubule, after exposure to increasing concentrations of: (Di) PM, (Dii) CIS or (Diii) DOX; n = 8 for all groups. White scale bars represent 50 µm; yellow scale bars in insets represent 10 µm. Data are mean + SEM; p < 0.001 (***) indicate significant differences in Mvh^+^ germ cells for treatment versus Control.

Expression of mouse vasa homologue (Mvh) was used to identify germ cells using immunohistochemistry (IHC), with seminiferous tubules of Control tissue lined by germ cells, many of which were proliferative, as shown by incorporation of bromo-2’-deoxyuridine (BrdU; Fig. 3A). In response to exposure to any of the three chemotherapeutic agents, there was a marked loss of germ cells from the seminiferous tubules (Fig. 3B,C). Proliferating Mvh^+^ /BrdU^+^ germ cells were still seen after exposure to the Low concentration of each drug, even amongst the small number of germ cells that remained after exposure to Low CIS or Low DOX (Fig. 3B). In contrast, after exposure to the High drug concentrations, no Mvh^+^/BrdU^+^ dividing germ cells were observed in the High CIS or DOX analysis (Fig. 3Cii,iii), and no remaining germ cells at all were seen after exposure to High PM (Fig. 3Ci). Counts of Mvh^+^ cells showed that all three chemotherapeutic drugs induced a significant reduction in germ cells after exposure to all concentrations, although this effect was less pronounced after exposure to Low PM (p < 0.001): (Fig. 3D). The difference between Control and treatment group germ cell numbers was as follows: Low PM 1.5-fold difference, Mid PM 20-fold difference; High PM no Mvh^+^ cells remaining; Low CIS 40-fold difference; Mid/High CIS 500- to 1000-fold difference; Low and Mid DOX 50-fold difference; and High DOX 100-fold difference. The majority of germ cells were seen to be proliferating (Mvh^+^/BrdU^+^), other than where the germ cell population had been reduced by 100-fold or more, where few if any proliferating germ cells remained (Fig. 3D).

**Figure 4:**
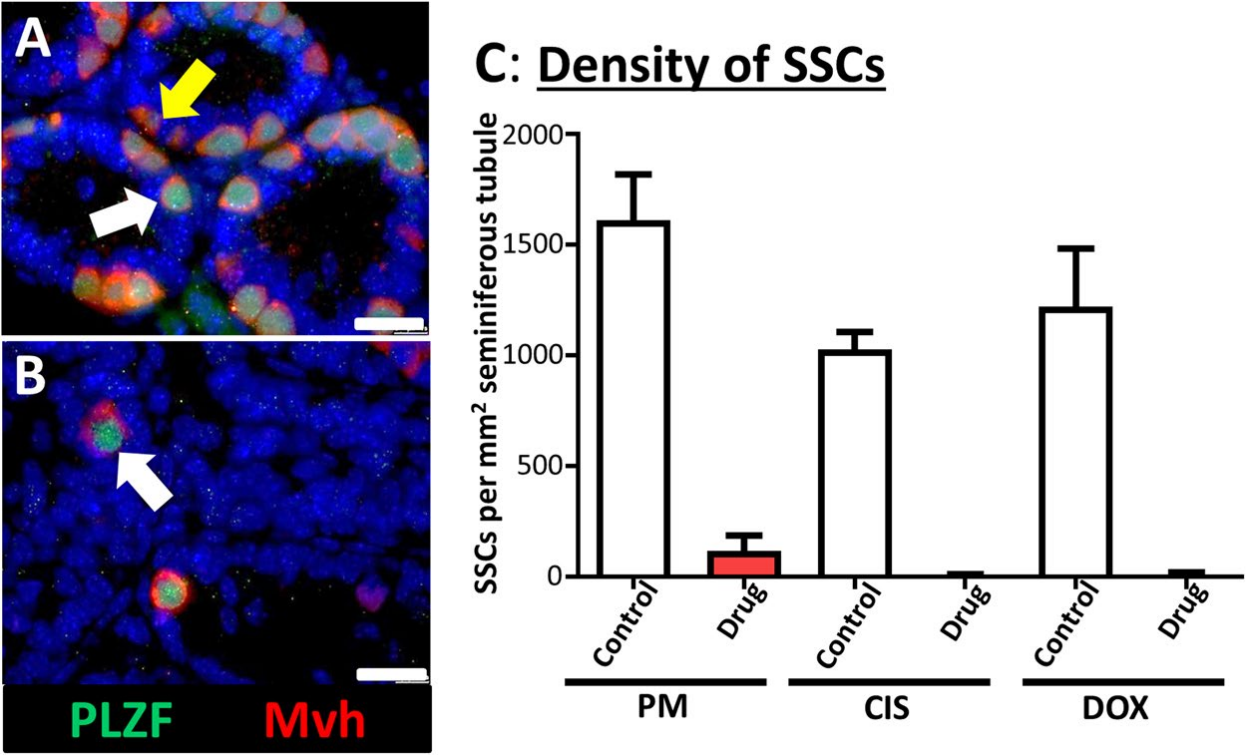
Exposure to phosphoramide mustard, cisplatin or doxorubicin results in loss of spermatogonial stem cells (SSCs). (**A,B**) Representative photomicrographs of cultured testis fragments from (**A**) Control and (**B**) treated (PM), with immunohistochemical localization of PLZF (green) to identify SSCs, and of Mvh (red) to identify all germ cells; sections are counterstained with DAPI (blue). White arrows indicate PLZF^+^/Mvh^+^ SSCs; yellow arrow indicates PLZF^−^/Mvh^+^ non-SSC germ cells. (**C**) Number of PLZF^+^ SSCs per mm^2^ of seminiferous tubule after exposure to Mid PM, Low CIS or Low DOX; n = 4–8. Scale bars represent 25 µm. Data are mean + SEM; p < 0.05 (*), p < 0.01 (**) for treatment versus Control.

Germ cells were then investigated further, to specifically examine SSCs, since this is the germ cell population required for the production of new germ cells and necessary for any subsequent repopulation of depleted seminiferous tubules. IHC was carried out for PLZF, expressed specifically in SSCs, after exposure to Mid PM, Low CIS or Low DOX: the Mid PM concentration was used due to the markedly higher survival of germ cells after Low PM compared to Low CIS or Low DOX exposure (Fig. 3). As with Mvh^+^ cells, few PLZF^+^ SSCs remained after exposure to PM, with only occasional SSC cells remaining after exposure to CIS or DOX (Fig. 4).

### Somatic cell density is unaffected by cyclophosphamide, cisplatin or doxorubicin

**Figure 5:**
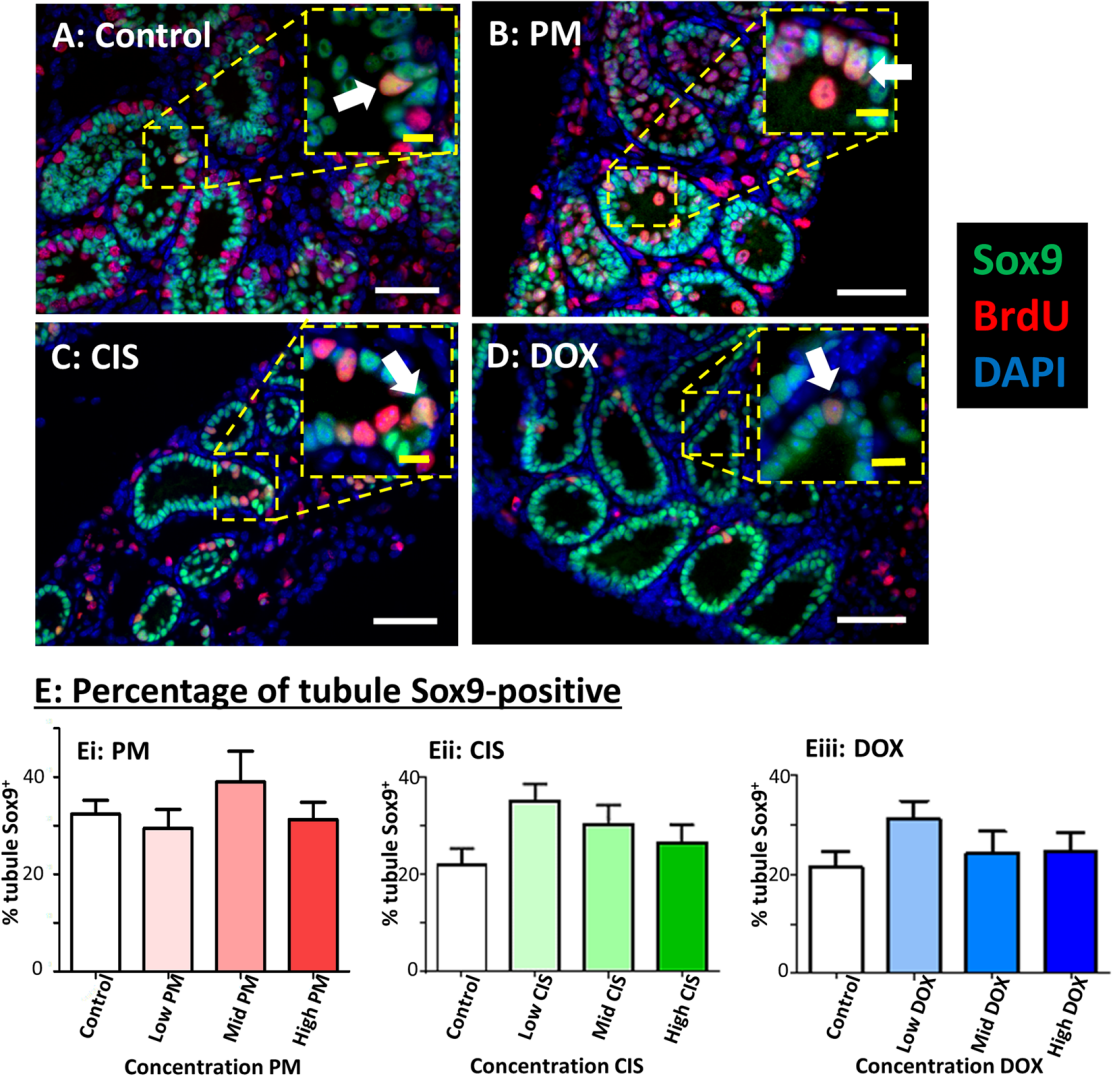
Sertoli cell density is unaffected by exposure to phosphoramide mustard, cisplatin or doxorubicin. (**A–D**) Representative photomicrographs of cultured testis fragments showing immunohistochemical localisation of Sox9 (green) and BrdU (red), counterstained with DAPI (blue). (**A**) Control cultured testis contains seminiferous tubules lined with Sox9^+^ Sertoli cells. (**B–D**) Tissue from High concentration of each of: (**B**) PM, (**C**) CIS or (**D**) DOX shows similar expression patterns to Control, including the occurrence of proliferating Sertoli cells. White arrows indicate Sox9^+^/BrdU^+^ proliferating Sertoli cells. (**E**) Percentage of tubule containing Sox9^+^ Sertoli cells after exposure to increasing concentrations of: (Ei) PM, (Eii) CIS or (Eiii) DOX; n = 8 in all groups except Low and High PM, where n = 7. White scale bars represent 50 µm, yellow scale bars in inset represent 10 µm. Data are mean + SEM.

Somatic Sertoli and interstitial cells were examined to see if they were also affected by any of the chemotherapy drugs. In contrast to the loss of germ cells, Sox9^+^ Sertoli cells were found lining the seminiferous tubules in all High drug-exposed tissues, as in Controls (Fig. 5A–D). Quantification of expression area showed no significant effect on the proportion of the tubules occupied by Sertoli cells on exposure to any of the drugs, even at High concentrations (Fig. 5E: (i) PM p = 0.54; (ii) CIS p = 0.63; (iii) DOX p = 0.93), with expression area shown to correlate closely with manual cell counts (p < 0.0001; Supplementary Figure S2). There is some Sertoli cell proliferation during early postnatal development and small numbers of Sox9^+^/BrdU^+^ proliferating Sertoli cells were seen in Control cultures (Fig. 5A inset) and in all treatment groups, even after exposure to High concentrations of each of the three drugs (Fig. 5B–D insets).

**Figure 6:**
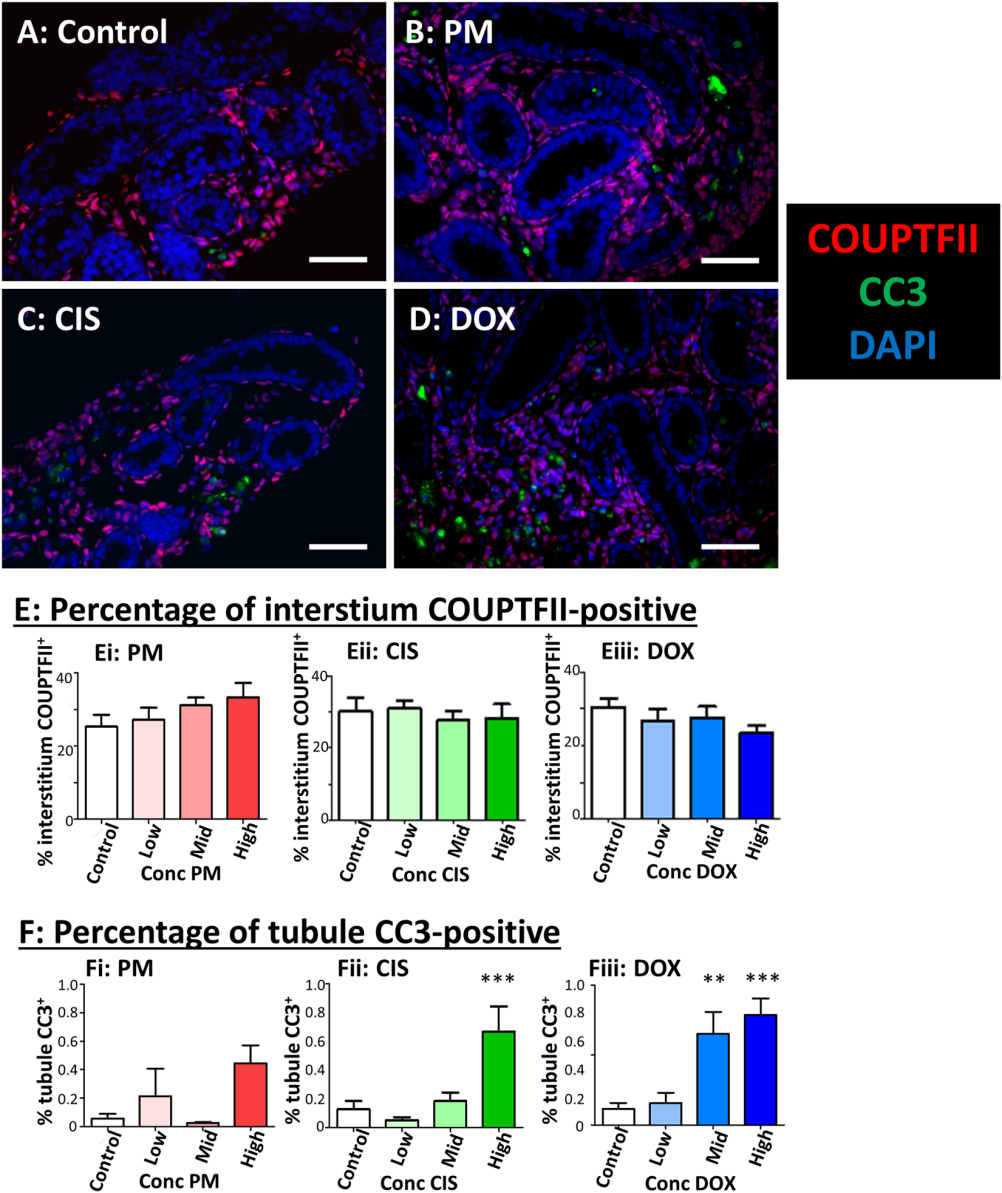
Apoptosis is increased, but interstitial cell density unaffected, by exposure to phosphoramide mustard, cisplatin or doxorubicin. (**A–D**) Representative photomicrographs of cultured testis fragments showing immunohistochemical localisation of COUPTFII (red) and CC3 (green), counterstained with DAPI (blue). (**A**) Control cultured testis shows COUPTFII expression in the majority of interstitial cells, with little evidence of apoptosis (CC3-expression). (**B–D**) Tissue from High concentration of each of: (**B**) PM, (**C**) CIS or (**D**) DOX shows similar COUPTFII expression pattern, alongside increasing evidence of apoptosis (CC3^+^ cells). (**E**) Percentage of interstitium positive for COUPTFII after exposure to increasing concentrations of: (Ei) PM, (Eii) CIS or (Eiii) DOX. (**F**) Percentage of seminiferous tubules positive for CC3 after exposure to increasing concentrations of: (Fi) PM, (Fii) CIS or (Fiii) DOX. E,F: n = 8 for all groups except High PM, where n = 7. Scale bars represent 50 µm. Data are mean + SEM; p < 0.01 (**), p < 0.001 (***) for treatment versus Control.

Interstitial cells were examined by determining expression of chicken ovalbumin upstream promoter transcription factor II (COUPTFII), expressed in the majority of interstitial cells in the neonatal mouse including the cells that will differentiate into adult Leydig stem cells^17^. The interstitium contained mainly COUPTFII^+^ cells, both in the Control cultured testis and after exposure to each of the chemotherapy drugs, even at High concentrations (Fig. 6A–D). Quantification of expression showed no effect of any drug on the percentage of the interstitial area containing COUPTFII^+^ interstitial cells (Fig. 6E: (i) PM p = 0.55; (ii) CIS p = 0.88; (iii) DOX p = 0.15).

### Increase in apoptosis and DNA damage in response to exposure to cyclophosphamide, cisplatin or doxorubicin

Apoptosis was examined using IHC for cleaved-Caspase-3 (CC3). At the end of the culture period, two days after exposure to the chemotherapy drugs had ended, there was relatively little expression of CC3 in testis exposed to Low concentrations of any of the three drugs (Fig. 6A–D). However, quantification of expression within seminiferous tubules showed a significant increase in apoptosis of around 5- to 6-fold after exposure to High CIS (p < 0.001) or to Mid or High DOX concentrations (p < 0.01 and 0.001 respectively; Fig. 6F). PM-treated tissue did not show significantly different levels of CC3 to Control at any concentration.

**Figure 7:**
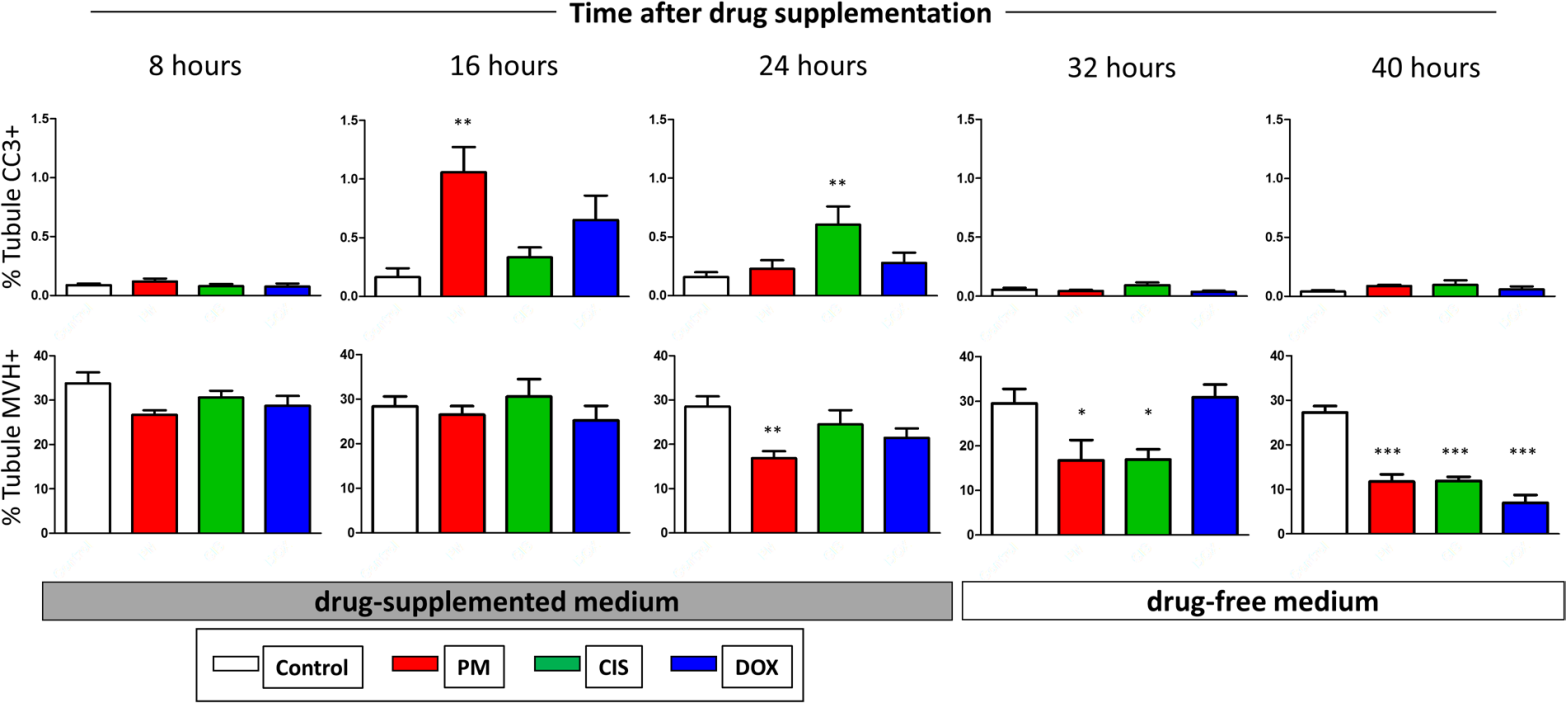
Time course analysis of apoptosis and germ cell loss in response to phosphoramide mustard, cisplatin or doxorubicin. Each chemotherapy drug induces a distinct pattern of apoptosis and germ cell loss immediately after drug exposure. Histogram of percentage of seminiferous tubules positive for CC3 (upper panel) and Mvh (lower panel) across eight-hourly intervals following the start of drug exposure, with tissue exposed to Mid PM, Low CIS or Low DOX; n = 4–9. Data are mean + SEM; p < 0.05 (*), p < 0.01 (**), p < 0.001 (***), for treatment versus its time-point Control.

Given that an increase in apoptosis might occur only as a more immediate response to the chemotherapy drugs, a time course analysis was undertaken. Testis fragments were cultured for 24 h in control medium, and then exposed to Mid PM, Low CIS or Low DOX: the Mid PM concentration was used due to the markedly higher survival of germ cells after Low PM compared to Low CIS or Low DOX exposure (Fig. 3). As in earlier experiments, drug exposure was for up to 24 h, after which tissue was moved back into drug-free medium. Tissue was removed from culture at 8 h intervals, for up to 40 h after the start of drug exposure. IHC was carried out to determine the level of apoptosis (CC3), and the number of germ cells (Mvh expression). Each of the three chemotherapy drugs induced a distinct pattern of response (Fig. 7): PM resulted in a significant increase in the amount of CC3 16 h after drug-exposure (p < 0.01) followed by a significant decrease in Mvh expression 8 h later, at the 24 h time point (p < 0.01); CIS resulted in a significant increase in the amount of CC3 after 24 h (p < 0.01) followed by a significant decrease in Mvh expression 8 h later, at the 32 h time point (p < 0.05); in contrast, there was no significant effect on CC3 at any time after DOX exposure. Here, Mvh expression was significantly decreased only at the 40 h time point (p < 0.001), but this decrease was not preceded by an increase in the amount of CC3. In case there was a shorter interval between an increase in the amount of CC3 and germ cell loss compared to the other drugs, levels of CC3 at 36 h following DOX-exposure was also investigated: no significant increase in CC3 was found at this time point, with the percentage of seminiferous tubule positive for CC3 as follows: mean ± sem of Control 0.12 ± 0.02 (n = 8) and DOX-exposed 0.21 ± 0.06 (n = 5); p = 0.14.

**Figure 8:**
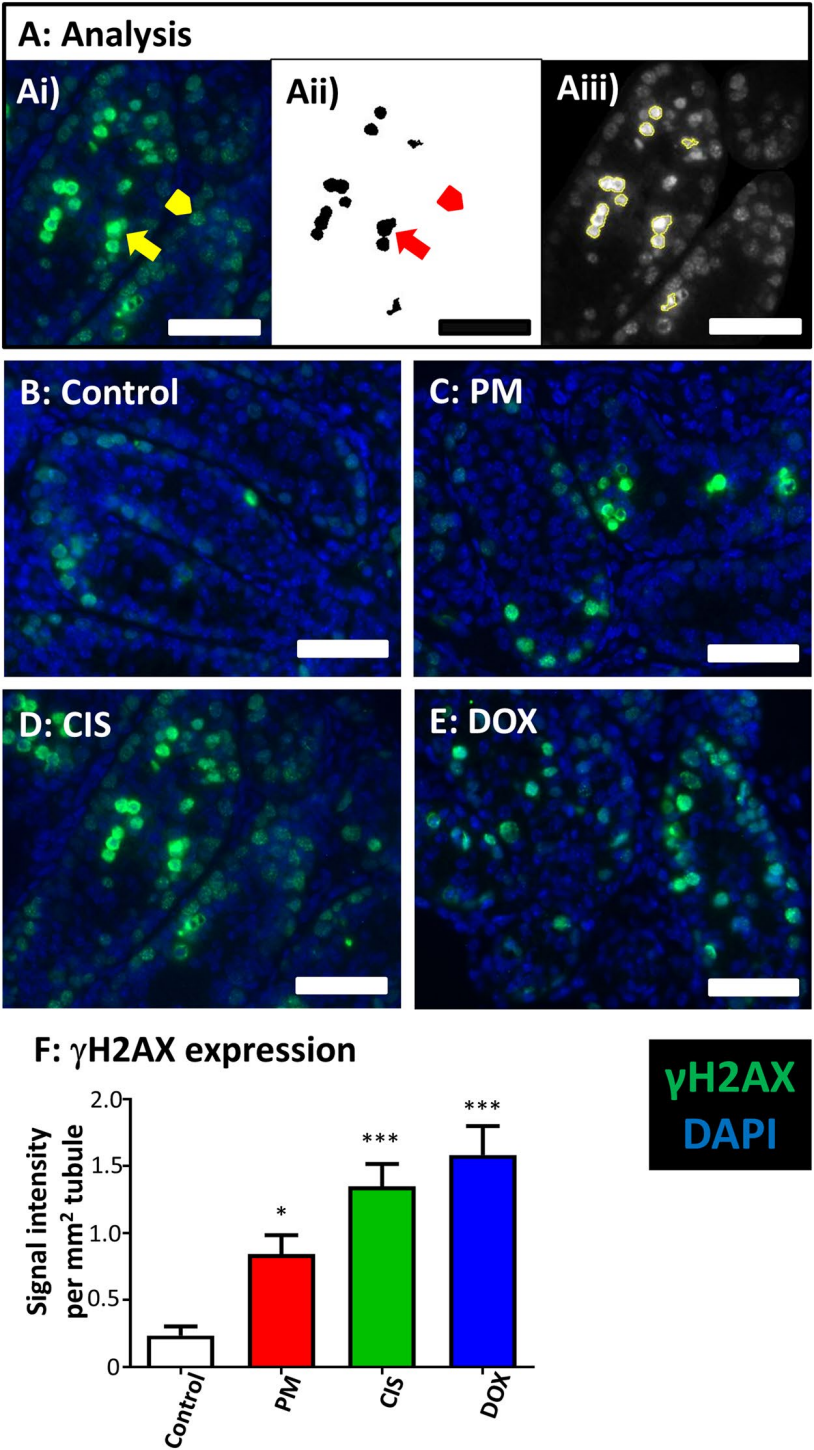
Exposure to phosphoramide mustard, cisplatin or doxorubicin leads to increased expression of phosphorylated H2AX. Representative photomicrographs of cultured testis fragments showing immunohistochemical localisation of γH2AX (green) of cultured testis tissue collected 16 h after the start of drug exposure, counterstained with DAPI (blue). (**A**) Analysis of γH2AX expression using ImageJ/Fiji software. (Ai) Photomicrograph showing cells with high intensity foci (arrow) and with low intensity foci (arrowhead) of γH2AX. (Aii) Black and white image of selected areas containing only cells with high intensity foci (arrow), after setting a high intensity threshold to exclude cells with low intensity foci (arrowhead) alongside a minimum particle size to exclude signals too small to be cells. (Aiii) Image showing the selected areas (black areas in Aii) from which pixel intensity is measured to obtain value of integrated density. (**B–E**) Representative images showing γH2AX expression from each group; (**B**) Control, (**C**) Mid PM, (**D**) Low CIS, (**E**) Low DOX. (**F**) Intensity of γH2AX expression normalised for tubule area; n = 6 in all groups. Scale bars represent 50 µm. Data are mean + SEM; p < 0.05 (*), p < 0.001 (***) for treatment versus Control.

DNA damage was then investigated by carrying out IHC for phosphorylated, γH2AX, analysing only the high intensity foci that occur in response to germ cell damage^18–20^. Preliminary experiments showed no significant change in γH2AX expression eight hours after the start of drug exposure (see Supplementary data S1), so tissue was then examined 16 h after exposure to PM, CIS or DOX (Fig. 8). Here, damage was evident after exposure to all three drugs (PM p < 0.05; CIS and DOX p < 0.01).

### Proliferation within seminiferous tubules is decreased on exposure to cisplatin or doxorubicin but not phosphoramide mustard

**Figure 9:**
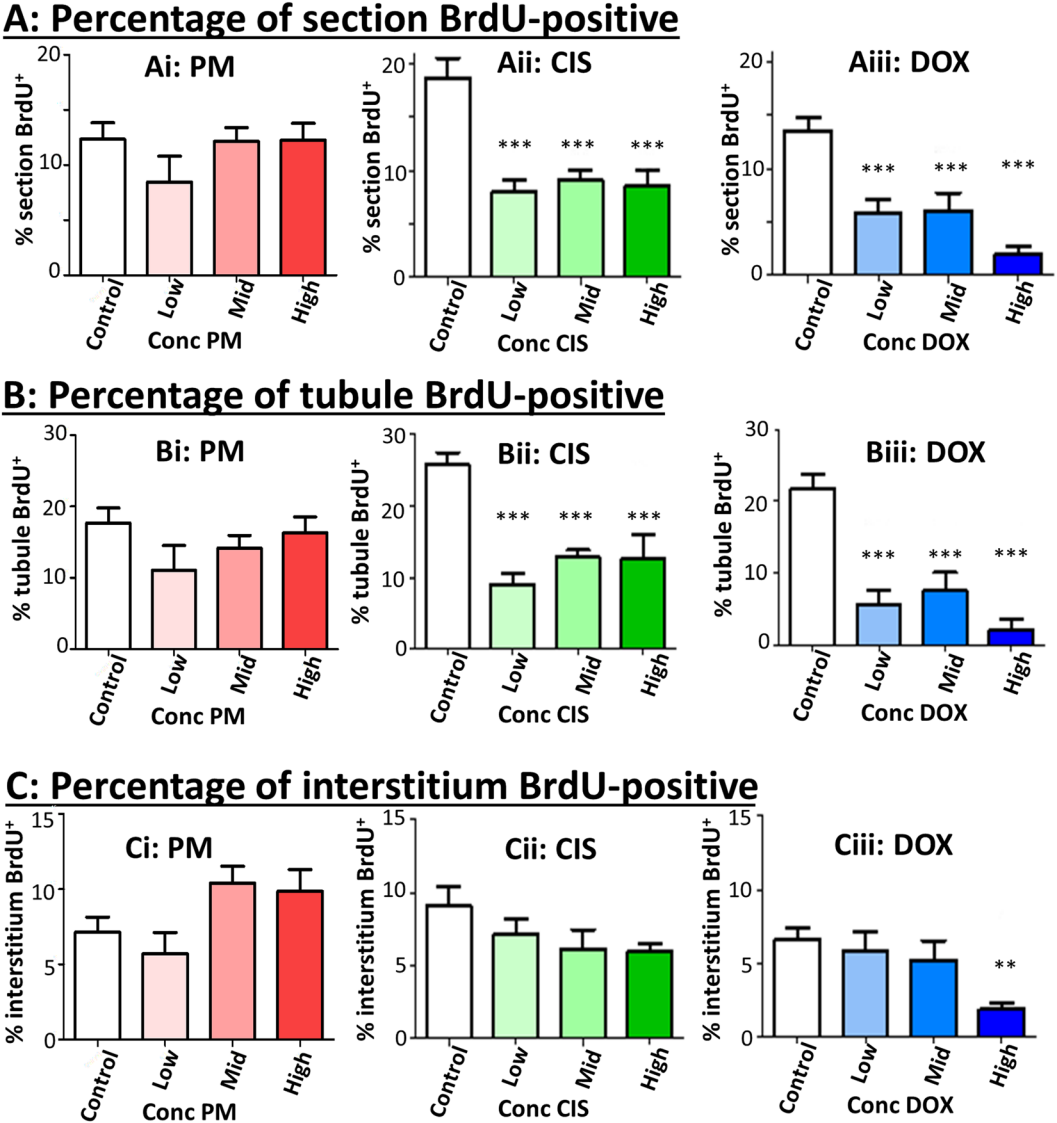
Proliferation decreases specifically within tubules after exposure to cisplatin or doxorubicin, but not to phosphoramide mustard. (**A**) Percentage of testis positive for BrdU. This was then examined according to (**B**) percentage of tubule positive for BrdU; and (**C**) percentage of interstitium positive for BrdU. (**A–C**) Quantification was determined after exposure to increasing concentrations of: (i) PM, (ii) CIS or (iii) DOX; n = 8 for all groups. Data are mean + SEM; p < 0.01 (**), p < 0.001 (***) for treatment versus Control.

BrdU was added to the medium for the final 24 h of culture in order to examine the proliferative capacity of the tissue following exposure to chemotherapeutics. BrdU incorporation decreased significantly after exposure to all concentrations of CIS (2-fold; p < 0.001 in each case) or DOX (2-fold reduction in response to Low or Mid DOX and 6- to 7-fold reduction in response to High DOX; p < 0.001 in each case): in contrast, there was no significant change in BrdU incorporation following PM exposure (p = 0.14; Fig. 9A). Separate examination of proliferation within the tubule (Fig. 9B) and interstitial (Fig. 9C) areas showed that the difference in proliferation in response to CIS or DOX exposure was primarily due to effects within the tubule, while there was no significant effect of CIS or DOX in the interstitium other than in the High DOX treatment group, which exhibited a 3.5-fold decrease in expression (p < 0.01). As with proliferation in the tissue as a whole, there was no effect of PM on proliferation within either the tubule or the interstitium.

## Discussion

It has long been recognised that chemotherapy treatment can have adverse effects on male reproduction, including impairment of subsequent fertility in patients with cancer who are treated prior to puberty, and hence before fully functional spermatogenesis^9,21^. These male childhood cancer patients are particularly important to consider, given that prepubertal boys are the one group in which fertility preservation strategies remain experimental at the present time^1^. There is, however, little information on the degree of gonadotoxicity or the specific actions of individual chemotherapy drugs. Here, we have examined the effect of three chemotherapy drugs that are commonly administered to childhood cancer patients; CYP (through administration of its active metabolite PM), CIS and DOX, using a prepubertal mouse *in vitro* system that has been shown to maintain normal morphological appearance of the seminiferous tubules and support the survival and proliferation of the germ and somatic cell compartments, with the Control cultured testis tissue similar to that *in vivo*^15^. Each of the three drugs induced a dramatic loss of germ cells when administered at clinically relevant concentrations, with little effect evident on the somatic cell component of the testis.

Culture medium was supplemented with PM, CIS or DOX, with effects of each drug determined across the range of concentrations that have been reported in patient serum. PM concentrations used were 0.02 (Low), 0.2 (Mid) or 2 (High) µg ml^−1^, equivalent to roughly 0.1 (Low), 1.0 (Mid) or 10 (High) μM, with from 0.1 to 10 μM previously found in patient serum^22,23^. Tissue was exposed to CIS at 0.1 (Low), 0.5 (Mid) or 1 (High) µg ml^−1^, with reported patient serum concentrations from 0.4 to 1 µg ml^−1^ ^24,25^. Finally, DOX was added to culture medium to produce concentrations of 0.05 (Low), 0.1 (Mid) or 0.5 (High) µg ml^−1^, with reports of 0.01 to 0.6 µg ml^−1^ in patient serum^13,26^. Whilst it is not possible to determine the concentrations of these drugs that occur within testicular tissue *in vivo*, these experiments here do allow comparison of the gonadotoxicity of the different drugs at concentrations relative to their circulating levels in patients.

The majority of work examining the effects of chemotherapy drugs on gonadal function in rodents have looked at the post-pubertal testis, with exposure to CYP, CIS and DOX all resulting in germ cell damage [for example^27–31^]. However, the adult testis, with fully functional spermatogenesis, is a very different, and more active cellular environment than the prepubertal testis, and there is no evidence that results obtained on the former tissue can be directly applied to the latter. In contrast to the more extensive literature on adult testis, few reports have been published on the effects of chemotherapy drugs on the prepubertal testis. This is despite the fact that prepubertal boys cannot yet be offered any fertility preservation method that has been shown to be successful in humans, with potential techniques such as testicular tissue transplantation or *in vitro* development of spermatozoa from spermatogonia currently achieved only in animal models^32,33^.

Results here showed that exposure to the Low concentration of PM (the active metabolite of CYP), had less of an effect on germ cell numbers than exposure to Low CIS or DOX (1.5 fold compared to 40- and 50-fold respectively), perhaps a surprising result given that the clinical literature indicates that treatment with alkylating agents in general, and cyclophosphamide in particular, has the strongest association with fertility problems in males^34,35^. However, for all three drugs, few germ cells remained after exposure to Mid or High concentrations. There has been little investigation into the effect of CYP on the prepubertal testis, with no effect reported after *in vivo* exposure of prepubertal rats^36^, while the testes of prepubertal mice who were administered CYP were not examined until after puberty^37^. To the best of the authors’ knowledge, there are no publications on laboratory investigations into the effects of CIS on the prepubertal testis. Finally, while DOX has previously been considered to be less gonadotoxic than CYP or CIS^14^, the gonadotoxic effects of DOX found on germ cells here are consistent with those in prepubertal rats *in vivo*^38,39^, and to a study in a prepubertal rat *in vitro* model using similar concentrations of DOX [0.04 and 0.1 µg ml^−1^;^40^].

The potential for germ cell repopulation of the seminiferous tubules after chemotherapy-induced loss will depend on the survival of proliferative germ cells, specifically those spermatogonia that are stem cells. SSCs are necessary both for self-renewal of the germ stem cell population and also for the cellular divisions that support the differentiation leading to complete spermatogenesis. A small number of Mvh^+^/BrdU^+^ proliferating germ cells did remain after Low CIS exposure, but importantly, PLZF-expressing SSCs were rarely seen after exposure to Low CIS concentrations. Damage induced by DOX-exposure was at least equivalent to CIS, with PLZF^+^ SSCs rarely seen even following exposure to Low DOX concentrations, while after exposure to High DOX, no BrdU^+^ proliferative germ cells remained. The situation after PM exposure was more complex: there was less effect on the germ cell population as a whole after exposure to lower concentrations, and more PLZF^+^ SSCs remained after Mid PM exposure, but in contrast, High PM exposure was the only treatment group in which no germ cells were found. Bearing in mind that experiments here used a short-term *in vitro* mouse model, our results highlight the importance of further work in this area, to directly determine whether SSCs are able to repopulate the seminiferous tubules following chemotherapy drug-induced damage, perhaps along with work using human tissue or non-human primate models. Overall, results here indicate that the longer term consequences of treatment with CYP are more pronounced at higher concentrations, and that DOX may be as, or even more damaging than CIS, with both of these drugs already inducing marked damage at relatively low concentrations. The effects of CYP and CIS tie in with clinical findings, but DOX has been considered to have more limited fertility consequences^9,21^. This could reflect the difficulty in determining the gonadotoxicity of individual drugs from clinical studies in which patients are treated with complex drug-combinations.

Whilst the present study clearly demonstrate a loss of spermatogonia in the prepubertal rodent testis following exposure to chemotherapeutics, including in the PLZF^+^ SSC population, there are a number of important considerations when extrapolating these findings to human. Similarities between the postnatal day 5 (pnd5) mouse and prepubertal human include the fact that the HPG axis is quiescent during this period of development and therefore there is no impact of gonadotrophins or of testosterone within the testis^5^. Whilst in both species, the germ cell population consists almost exclusively of spermatogonia^4^, the spermatogonial sub-populations differ. Importantly, in rodents, the A_single_ spermatogonia can perform a dual function by acting either as stem cell or progenitor, whilst in the human these functions are performed by the A_dark_ and A_pale_ spermatogonial populations respectively^16^. As such, it is important to bear in mind that this could result in differences in sensitivity to chemotherapeutics and also in the potential for subsequent recovery. Another important consideration regarding species differences in germ cells during this period of development is the rate of spermatogonial proliferation. Whilst in rodents there is a relatively high proliferation at pnd5, as demonstrated in the present study, the rate of prepubertal spermatogonial proliferation in humans and non-human primates is relatively low in comparison^4,8,41^. This is of particular relevance in relation to chemotherapy exposures, which often target mitotically active cells.

At the end of the four day culture period, 48 h after the drug exposure period had ended, there was little evidence of apoptosis, with significant increases in CC3 expression seen only after exposure to High CIS, or to Mid or High DOX, and with no significant increase found after PM exposure. Apoptosis was therefore examined in a time-course analysis at 8-h intervals following exposure to each drug, with results showing a difference in the response of the prepubertal testis to each of the three drugs. After exposure to PM or CIS, there was a similar pattern of a significant increase in apoptosis followed eight hours later by a significant decrease in germ cells, although the precise timing of this response differed between the two drugs, occurring earlier following exposure to PM than to CIS. In contrast, no significant increase in CC3 was seen at any time point after DOX-exposure, despite the significant decrease in germ cells first seen 40 h after the start of drug-exposure. The lack of a significant increase in CC3-expression in response to DOX could have been because increased expression occurred within the 8 h interval of the time course analysis; however, there was also no effect on CC3 expression midway through the time interval immediately before the significant effect on germ cells, 36 h after the start of DOX-exposure. Given the lack of any increase in CC3 expression in response to DOX exposure, DNA damage was investigated by examining expression of phosphorylated H2AX. Phosphorylation of H2AX is one of the earliest responses to double stranded DNA breaks (DSBs), with immunofluorescence analysis of γH2AX showing the presence of discrete foci in cell nuclei that have undergone DSBs^42^. The analysis here investigated only the intense foci of expression that are an indicator of germ cell DNA damage^18–20^, due to the occurrence of low intensity foci in healthy male germ cells^18,43^. Results showed a significant, marked increase in such foci in response to all three drugs after 16 h, with DOX exposure leading to the largest increase: CYP and CIS have previously been shown to result in an increase in γH2AX in a mouse spermatogonial cell line^20^. It remains to be seen if the DNA damage resulting from DOX exposure leads to apoptosis; or whether the germ cell loss may be the result of a different cell death pathway: DOX-induced cardiotoxicity has been shown to result in autophagy^44^, with senescence reported in cell lines^45,46^.

Despite the marked loss of germ cells, no significant effect was found on either Sertoli (area of Sox9 expression) or interstitial (area of COUPTFII expression) cell density. It is possible that more subtle damage has been induced in either/both somatic cell population(s), the effects of which would not become apparent until later. Downstream effects on the somatic cell population of the testis would be expected following germ cell loss: for example, absence of germ cells has been shown to alter Sertoli cell gene expression^47,48^. Alternatively, Sertoli cell damage following repeated administration of CIS to adult mice has been shown to result in long-term spermatogenesis problems despite the survival of stem germ cells^49^.

Effects of the chemotherapy drugs on proliferation rate in the somatic populations was determined by adding BrdU into the culture medium during the last 24 h of culture. There was no effect on proliferation of interstitial cells following exposure to any of the drugs (except High DOX). However, there was a highly significant and marked decrease in BrdU^+^ cells within the seminiferous tubules in response to even Low concentrations of CIS or DOX, while no such effect was found in response to PM exposure. Given the relatively low rate of proliferation and stable density of Sertoli cells in Controls at this stage, the reduction in proliferation is likely to reflect the loss of proliferating germ cells. In the human, Sertoli cell proliferation occurs only during the neonatal period and following puberty, in contrast to the low level of proliferation that occurs throughout pre-puberty in the mouse. Findings here are in contrast with the effect of irradiation on the somatic compartment, which leads to a reduction in number of Sertoli cells and increase in apoptotis^50,51^. Moreover further studies are advisable in order to assess whether the production of key growth factors and other cytokines are affected by chemotherapy exposure, as shown after irradiation^52^.

In summary, the work here, using an *in vitro* model of the mouse prepubertal testis, shows that the three chemotherapy drugs CYP (through use of its active metabolite PM), CIS and DOX each induced a rapid and marked decrease in the germ cell population after exposure to clinically relevant concentrations, and that this includes a decrease in the stem germ cell population. CIS and DOX exposure led to a marked drop in germ cell numbers even at the lower range of concentrations found in patient serum, while the effect of PM was less pronounced until higher concentrations were used, although High PM was the only treatment that led to a complete disappearance of germ cells. The total loss of germ cells in response to High PM alongside the near-disappearance of PLZF^+^ SSCs after exposure to Low concentrations of CIS or DOX, indicate that all three drugs may produce long-term reproductive damage. While the effects of CYP/PM and CIS are consistent with effects on fertility described in the clinical literature, results here indicate that DOX may have a similar level of gonadotoxicity as CYP and CIS. This work used prepubertal mouse testis as the experimental model, and whilst differences between rodent and primate testicular development during this period prevent direct translation of the findings into human, the present study has identified potentially important information on the effects of chemotherapy expsoures that may be used to inform targeted studies in the prepubertal human or non-human primate testis, for which tissue is scarce and experimental models are currently lacking.

## Methods

### Animals

All work was approved by the University of Edinburgh’s Local Ethical Review Committee and carried out in accordance with UK home office regulations. C57Bl/J6 mice were housed in a 14 h light:10 h dark photoperiod, with access to food and water *ad libitum*.

### Tissue Culture

Testis tissue was cultured as in Lopes and co-authors^15^. Testes from a minimum of two pnd5 mice per culture run were dissected into Leibovitz L-15 dissection medium (Invitrogen, UK) supplemented with 3 mg ml^−1^ bovine serum albumin (BSA; Sigma-Aldrich Ltd, UK) at 37 °C and then further dissected into pieces of approximately 0.5 mm^3^. Each piece of testis was transferred onto Whatman Nucleopore membrane (Camlab Ltd, UK) floating on 1 ml of α-MEM medium (Invitrogen, UK) supplemented with 10% Knockout Replacement Serum (Invitrogen, UK), within a 24-well plate (Greiner Bio-one, UK), and incubated at 37 °C, 5% CO_2_. Cultures were left for 24 h (Day 1).

On Day 2 of culture, medium was supplemented with PM (Niomech, Germany), CIS (Sigma Aldrich, UK) or DOX (Sigma Aldrich, UK), with three concentrations per drug chosen to cover the range of reported serum levels in patients [PM:^22,23^; CIS:^24,25^; DOX:^13,26^], and with the effect of each drug compared to Control cultures that were maintained in drug-free medium throughout. Drugs were dissolved in culture medium to produce final concentrations as follows: PM - 0.02 µg ml^−1^ (Low PM), 0.2 µg ml^−1^ (Mid PM), or 2 µg ml^−1^ (High PM); CIS - 0.1 µg ml^−1^ (Low CIS), 0.5 µg ml^−1^ (Mid CIS) or 1 µg ml^−1^ (High CIS); and DOX - 0.05 µg ml^−1^ (Low DOX), 0.1 µg ml^−1^ (Mid DOX) or 0.5 µg ml^−1^ (High DOX) respectively. Due to its volatility, cultures containing PM were placed in separate culture plates and kept apart from other plates in the incubator^53^. On Day 3 of culture, membranes were transferred into fresh control medium. On the final day of culture (Day 4), medium was additionally supplemented with 15 µg ml^−1^ 5- BrdU (Sigma Aldrich Ltd, UK) for subsequent determination of cell proliferation. Twenty-four hours later, tissues were fixed for 90 mins in 10% neutral buffered formalin solution (Sigma Aldrich Ltd, UK) for IHC or for 60 mins in Bouin’s fluid for histological examination, set in 3% agar (Sigma Aldrich Ltd, UK) and embedded in paraffin wax. For each drug, a minimum of 7 testis fragments from each concentration were examined, obtained from 2–3 culture runs.

For examination of spermatogonial stem cells (SSC), and for the time-course analysis, tissue was cultured either in control medium or exposed to the lowest drug concentration that earlier experiments had shown to result in a significant germ cell loss, namely Mid PM, Low CIS or Low DOX (Fig. 3). PLZF (marker of SSC) expression was examined after the four day culture period, as above. For the time-course analysis, tissue was removed from culture at eight-hourly intervals following the start of drug administration, and then processed as above. For each time point, 4–9 testis fragments were analysed, taken from a minimum of 2 culture runs, with specific sample sizes as follows: 8 hours - n = 4–5; 16 hours - n = 5–7; 24 hours - n = 5–7; 32 hours - n = 6–9; 40 hours - n = 5–8. Gamma-H2AX (marker of double-strand DNA breaks) expression was examined after 16 hours only; n = 6 for all.

### Histology

Wax blocks were sectioned at 5 µm, and either taken for IHC (see below) or photomicrographs taken of haematoxylin and eosin-stained sections (DMLB Leica microscope, Leica Microsystems Ltd, UK) and used for morphological examination by a blind-to-treatment assessor using ImageJ software. In each section, the number of seminiferous tubules that lacked visible germ cells on the basement membrane (Sertoli cell-only tubules) was noted, and the diameter of every spherical tubule was measured.

### Immunohistochemistry

IHC reactions were carried out on one section from the middle of each testis fragment. Washes in phosphate-buffered saline (Fisher Scientific UK Ltd, UK) with 0.1% Triton X (PBSTx) were performed between each step. Antigen retrieval was performed in 0.01 M citrate buffer (pH6, Sigma Aldrich Ltd, UK), followed by blocking step with 20% normal goat serum diluted in PBSTx and 5% w/v BSA for 1 h at RT. Slides were incubated with primary antibodies (Table 1) overnight at 4 °C in a humidified environment followed by incubation with appropriate secondary antibody and visualisation reagent all at 1:200 dilution (Table 1): specificities of expression patterns were tested by omission of primary or secondary antibodies. Details of all double IHCs are given in Table 2. Counterstaining was with 4,6-Diamidino-2-phenylindole (DAPI; Invitrogen, UK) at 1:5000 for 10 min and slides were then mounted with Vectashield hard-set mounting medium (Vector Laboratories, USA).

**Table 1:** Antibodies and conditions for immunohistochemistry.

Antibody	Catalogue^#^	Species	Dilution	Secondary Antibody	Visualisation
BrdU^1^	ab6326	Rat	1:500	GARat-b^5^; GARat-594^6^	Str-488^5^
CC3^2^	Asp175	Rabbit	1:500	GAR-b^5^	Str-488^5^
COUPTFII^3^	PP-H7147-00	Mouse	1:200	GAM-568^7^	N/A
γH2AX (phospho S139)^1^	ab22551	Mouse	1:200	GAM-488^8^	N/A
Mvh^1^	ab27591	Mouse	1:100	GAM-568^7^	N/A
PLZF^1^	ab189849	Rabbit	1:3000	GAR-488^7^	N/A
Sox9^4^	AB5535	Rabbit	1:500	GARat-594^6^	N/A

Abbreviations:

GAM-488: Alexafluor Goat anti-mouse IgG 488 nm.

GAM-568: Alexafluor Goat anti-mouse IgG_1_ 568 nm.

GARat-594: Alexafluor Goat anti-rat IgG_1_ 594 nm.

GAR-488: Alexafluor Goat anti-rabbit IgG 488 nm.

GAR-b: Goat anti-rabbit biotinylated.

GARat-b: Goat anti-rat biotinylated.

Companies: ^1^Abcam, UK; ^2^Cell Signalling Technology, USA; ^3^R&D systems, USA; ^4^Millipore, UK; ^5^Vector Laboratories, USA; ^6^Invitrogen, UK; ^7^Life Technologies, UK; ^8^Molecular Probes, Leiden, The Netherlands.

**Table 2:** Double immunohistochemistry protocol.

Antigen 1	Dilution	Secondary Antibody	Visualis- ation	Antigen 2	Dilution	Secondary Antibody	Visualis-ation
CC3^1^	1:500	GAR-b^5^	Str-488^8^	Mvh^3^	1:100	GAM-568^6^	N/A
COUPTFII^2^	1:200	GAM-568^6^	N/A	CC3^1^	1:500	GAR-b^5^	Str-488^8^
Mvh^3^	1:100	GAM-568^6^	N/A	BrdU^3^	1:500	GARat-b^5^	Str-488^8^
Sox9^4^	1:500	GARat-594^7^	N/A	BrdU^3^	1:500	GARat-b^5^	Str-488^8^

Abbreviations:

GAM-488: Alexafluor Goat anti-mouse IgG 488 nm.

GAM-568: Alexafluor Goat anti-mouse IgG_1_ 56 nm.

GARat-594: Alexafluor Goat anti-rat IgG_1_ 594 nm.

GAR-488: Alexafluor Goat anti-rabbit IgG 488 nm.

GAR-b: Goat anti-rabbit biotinylated.

GARat-b: Goat anti-rat biotinylated.

Str-488: Streptavidin 488.

Companies:

^1^Cell Signalling Technology, USA; ^2^R&D systems, USA; ^3^Abcam, UK; ^4^Millipore, UK; ^5^Vector Laboratories, USA; ^6^Life Technologies, UK; ^7^Invitrogen, UK; ^8^Molecular Probes, Leiden, The Netherlands.

### Image Acquisition and Analysis

Images were taken using a Leica DM5500B microscope with a DFC360FX camera. Image analysis was performed using ImageJ, with the assessor blind to treatment. For analysis of germ cells and proliferating germ cells, Mvh^+^/BrdU^−^, Mvh^+^/BrdU^+^ cells and PLZF^+^ cells were counted manually. For all other antibodies excepting γH2AX, each fluorophore area assessed was measured as a percentage of the section, tubule or interstitial area, as appropriate: this semi-automated system was verified by comparison with manual cell counting (Supplementary Figure S2), similar to results shown previously^15^. Analysis of phosphorylated H2AX expression examined cells with high intensity expression only (Fig. 8A), due to the appearance of low intensity γH2AX foci in healthy male germ cells^18,43^, in contrast to the high intensity foci seen in damaged male germ cells^18–20^. From an initial image showing cells with low and high intensity foci (Fig. 8Ai), a high threshold was set in order to analyse only the cells with high intensity foci, alongside setting a minimum particle size to exclude any signal too small to be a cell. By calculating the intensity values of the pixels only in those areas selected in the grayscale image (Fig. 8Aii), the integrated density of γH2AX expression was determined (Fig. 8Aiii), with results then normalized for tubule area.

### Statistical analysis

For all analyses other than that of the time-course experiment, Minitab software was used. Statistical significance of treatment was determined using one-way ANOVA with a general linear mixed effect model to take culture run into account. This was followed by Dunnett’s multiple comparisons to compare effects of different concentrations with Control. Data from the time-course study were analysed using GraphPad Prism. Here, for normally distributed data, one-way ANOVA was performed followed by Dunnett’s *post hoc* test to determine statistical significance between Control and different drug concentrations; for data that were not normally distributed, Kruskal-Wallis was performed followed by Dunn’s *post hoc* test to determine statistical significance between Control and different drug concentrations. All results are given as mean ± SEM, with results considered statistically significant where p < 0.05.

### Data availability

All datasets generated and analysed during the current study are available: datasets used for specific figures are in Supplementary data S1; other datasets are available from the corresponding author on reasonable request.

## Electronic supplementary material

Supplementary Data S2

Supplementary Figure S2
