# Supplementary material for: Chemotherapy drugs cyclophosphamide, cisplatin and doxorubicin induce germ cell loss in an in vitro model of the prepubertal testis
Source: Sci Rep. 2018 Jan 29;8:1773. doi: 10.1038/s41598-018-19761-9 (PMC5788858; doi:10.1038/s41598-018-19761-9)
Supplement: Supplementary file 2 — Supplementary Figure S2 [file 41598_2018_19761_MOESM2_ESM.doc]

**Chemotherapy drugs cyclophosphamide, cisplatin and doxorubicin induce germ cell loss in an in vitro model of the prepubertal testis.**

**Authors: E Smart, F Lopes, S Rice, B Nagy, RA Anderson, RT Mitchell, N Spears**

**Supplementary Figure S2.**


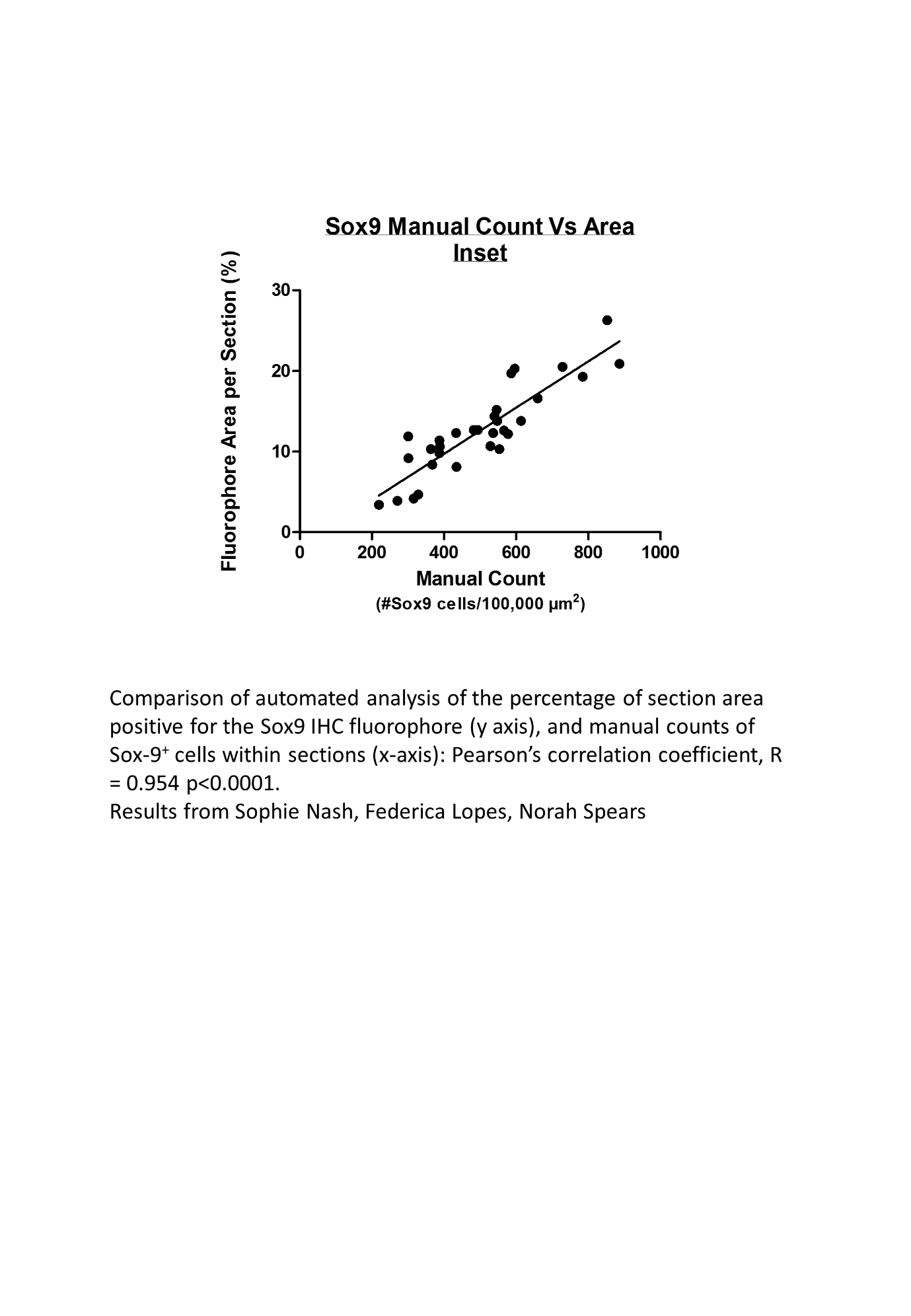
**Supplementary Figure S2.**

**Comparison of automated and manual counting of immunofluorescence images.**
